# Abdominal CT Does Not Improve Outcome for Children with Suspected Acute Appendicitis

**DOI:** 10.5811/westjem.2015.10.25576

**Published:** 2015-12-10

**Authors:** Danielle I. Miano, Renee M. Silvis, Jill M. Popp, Marvin C. Culbertson, Brendan Campbell, Sharon R. Smith

**Affiliations:** * Connecticut Children’s Medical Center, Department of Research, Hartford, Connecticut; † Connecticut Children’s Medical Center, Department of Surgery, Hartford, Connecticut; ‡ Connecticut Children’s Medical Center, Department of Emergency Medicine, Hartford, Connecticut; § University of Connecticut School of Medicine, Farmington, Connecticut

## Abstract

**Introduction:**

Acute appendicitis in children is a clinical diagnosis, which often requires preoperative confirmation with either ultrasound (US) or computed tomography (CT) studies. CTs expose children to radiation, which may increase the lifetime risk of developing malignancy. US in the pediatric population with appropriate clinical follow up and serial exam may be an effective diagnostic modality for many children without incurring the risk of radiation. The objective of the study was to compare the rate of appendiceal rupture and negative appendectomies between children with and without abdominal CTs; and to evaluate the same outcomes for children with and without USs to determine if there were any associations between imaging modalities and outcomes.

**Methods:**

We conducted a retrospective chart review including emergency department (ED) and inpatient records from 1/1/2009–2/31/2010 and included patients with suspected acute appendicitis.

**Results:**

1,493 children, aged less than one year to 20 years, were identified in the ED with suspected appendicitis. These patients presented with abdominal pain who had either a surgical consult or an abdominal imaging study to evaluate for appendicitis, or were transferred from an outside hospital or primary care physician office with the stated suspicion of acute appendicitis. Of these patients, 739 were sent home following evaluation in the ED and did not return within the subsequent two weeks and were therefore presumed not to have appendicitis. A total of 754 were admitted and form the study population, of which 20% received a CT, 53% US, and 8% received both. Of these 57%, 95% CI [53.5,60.5] had pathology-proven appendicitis. Appendicitis rates were similar for children with a CT (57%, 95% CI [49.6,64.4]) compared to those without (57%, 95% CI [52.9,61.0]). Children with perforation were similar between those with a CT (18%, 95% CI [12.3,23.7]) and those without (13%, 95% CI [10.3,15.7]). The proportion of children with a negative appendectomy was similar in both groups: CT (7%, 95% CI [2.1,11.9]), US (8%, 95% CI [4.7,11.3]) and neither (12%, 95% CI [5.9,18.1]).

**Conclusion:**

In this uncontrolled study, the accuracy of preoperative diagnosis of appendicitis and the incidence of pathology-proven perforation appendix were similar for children with suspected acute appendicitis whether they had CT, US or neither imaging, in conjunction with surgical consult. The imaging modality of CT was not associated with better outcomes for children presenting to the ED with suspected appendicitis.

## INTRODUCTION

Acute appendicitis in the pediatric population remains one of the most common surgical emergencies.^1^ The risk of developing appendicitis over the course of a lifetime is 7% in females, and 9% in males.^2^,^3^ In the United States, there are more than 70,000 appendectomies performed on pediatric patients 3–18 years old each year.^4^ Despite its high incidence, appendicitis may be challenging to diagnose due to the overlap of symptoms with other acute abdominal conditions or atypical presenting symptoms.^5^–^8^ Timely diagnosis and treatment of acute appendicitis is important to prevent complications such as a perforated appendix.^9^ Radiographic imaging studies such as ultrasound (US) and computed tomography (CT) are frequently ordered to aid in the diagnosis of patients who present with symptoms consistent with acute appendicitis.

With the advent of the helical CT study, physicians can rapidly obtain a three-dimensional view of the appendix and abdominal region. Image capture is estimated to take less than one second, which diminishes the need to anesthetize the child before a CT.^10^ The high image resolution, diagnostic accuracy, and convenience of a CT study have been contributing factors associated with its frequent use as a diagnostic tool.^11^

As the utilization of CT studies has increased over recent decades, the risks associated with varying doses of ionizing radiation have been estimated using data from atomic bomb survivors.^10^–^12^ For children younger than 15 years, the estimated risk of dying from a radiation-induced malignancy ranges from 0.07%–0.10%, with children in the lower ages having a higher estimated risk.^13^ In a recent retrospective cohort study the estimated risk for children younger than 15 developing leukemia and brain tumors tripled if a child had undergone more than two CTs.^14^ Additionally, children are more susceptible to the effects of ionizing radiation because they have a higher rate of cell divisions in developing tissues. Their younger age also leaves more years of life in which a radiation-induced malignancy may develop.^15^ Brenner and colleagues estimated that approximately one million children per year are unnecessarily exposed to harmful radiation from CTs.^10^

Despite the increased use of CTs, additional imaging studies may not improve the accuracy of the preoperative diagnosis of acute appendicitis.^16^,^17^ Flum and colleagues found that the increased use of CT and US studies have not impacted the population-level rate of negative appendectomy.^18^,^19^ In addition, a recent retrospective study found there was no increase in negative appendectomy or perforation rate following the implementation of a multi-disciplinary diagnostic protocol which used US as the initial diagnostic imaging study.^20^

The aim of this study was to determine if a correlation exists between children who received diagnostic imaging (CT and/or US) and two clinical outcomes: the rate of perforation and negative appendectomy. The hypothesis was that diagnostic imaging does not improve clinical outcomes for children with suspected acute appendicitis.

## METHODS

We conducted this study at an urban, tertiary-care children’s hospital with over 50,000 emergency department (ED) visits per year. Approval was obtained from the institutional review board to conduct a retrospective chart review of pediatric emergency medicine (PEM) patients. We screened ED and inpatient electronic medical records (EMR) from January 1, 2009 through December 31, 2010 to identify patients who presented to the ED with suspected acute appendicitis. The ED EMR system is PulseCheck version 5.0 Picis Inc., and the radiology EMR system is Phillips I-Site Version 3.6. Demographic data were automatically exported from the ED EMR into an excel spreadsheet. Additional data that could not be exported were hand entered onto this spreadsheet. The data from inpatient and radiology EMRs were hand entered onto the same spreadsheet. Initial screening to meet the primary inclusion criterion of suspected acute appendicitis was done by the primary author who had two years of research experience in this ED.

**Figure 1:**
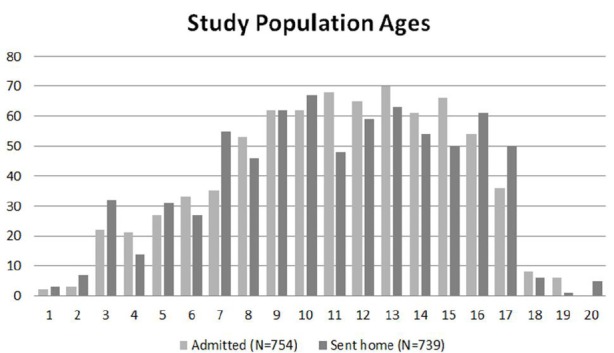
Histogram of study population ages (N=754) compared to population sent home (N=739).

Inclusion criteria were all patients who met the following definition for suspected acute appendicitis, presented to the ED with acute abdominal pain who had either a surgical consult or an abdominal imaging study (ultrasound, CT) or presented to and were transferred from an outside hospital or primary care physician office with the stated suspicion of acute appendicitis. The decision to obtain imaging and the type of imaging was determined by the board-certified pediatric emergency physician, certified pediatric advanced practitioner in the ED, or the board-certified pediatric surgeon. Physicians in training did not make imaging decisions without first consulting their respective attending physicians. We included all patients aged birth to 20 years who met the pre-determined definition for suspected appendicitis (Figure 1). Patients were excluded if they eloped from the ED, left against medical advice, or if they had a previous appendectomy. Data collected from the ED EMR included the following: demographics, location of initial presentation, chief complaint, results and location of radiographic imaging (plain film, US, CT), disposition from ED, previous surgeries, chronic medical conditions, antibiotics administered, duration of pain, and inpatient length of stay. Imaging studies were classified as positive for appendicitis if the radiology report stated “enlarged or thickened appendix,” “consistent with acute appendicitis” or “consider appendicitis.” Imaging studies were classified as negative if the radiology report stated “appendix not visualized,” or “normal appearing appendix.”

Inpatient charts for children who were admitted for observation or transferred to the operating room (OR) were evaluated for surgical documentation of appendectomy. We verified diagnosis of appendicitis by review of the pathology report. Appendicitis was defined as a pathology report that stated “acute appendicitis,” “gangrenous appendix,” or if any appendiceal inflammatory changes were documented within the report. Negative appendectomy was defined as an appendix with no inflammatory changes. Appendiceal perforation was determined by reviewing both the operative and pathology report. For purposes of analysis, we categorized patients sent home and not known to return as not having appendicitis (if true).

This retrospective chart review followed the methods outlined by Kaji and Schriger.^21^ All demographic data, chief complaints, dispositions, and lengths of stay were automatically exported into an Excel spread sheet. Additional data columns were created for entry of other data and used as the data collection form. Undergraduate research assistants, trained and participating in a for-credit research course, abstracted location of original presentation (children’s hospital or community hospital), location and type of all radiology studies ( plain radiographs, USs, CTs), reported results of radiographic studies, if done at outside hospital, based on scanned copies of interpretation in the EMR or (if not available) reported results documented by the physician provider, antibiotic medications given, and duration of reported pain from history of present illness section. All data abstracted was reviewed for correctness and accuracy by the primary and secondary authors. Any discrepancies were reviewed by both authors and final data were determined, after they verified the accuracy of all data entered. No data analyses were done until the final, cleaned database was completed.

We compared the data in two phases. The first comparison grouped children who had a pre-operative abdominal CT and children without a CT to rate of pathology-proven diagnosis of appendicitis, rate of perforation, and rate of negative appendectomy using chi-square analyses. Patients who had an abdominal CT before surgery, regardless of any other imaging, were included in this group. Secondly, we divided the cohort into three groups of patients: children who had an abdominal CT before surgery, children who had a pre-operative abdominal US, and children who had no imaging. Chi-square analyses were used to compare rate of pathology proven appendicitis, rate of perforation, and rate of negative appendectomy among these three groups. We conducted all analyses using SPSS 17.0.

## RESULTS

**Figure 2:**
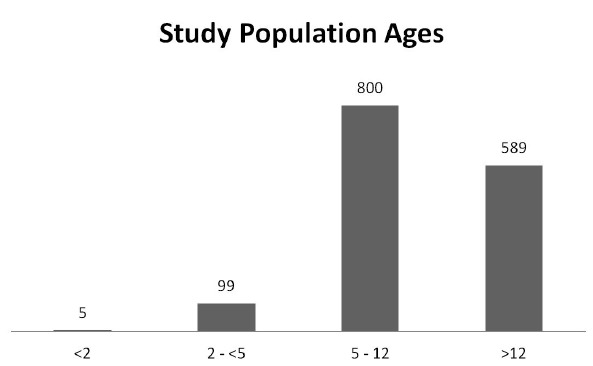
Histogram of study population ages (N=754).

Between January 1, 2009 through December 31, 2010, 1,493 children presented to the pediatric ED with suspected acute appendicitis, with a mean age of 11 years (SD=4) (Figure 2). Reported ethnicities were 54% Caucasian, 25% Hispanic, 10% African-American, and 50% were female (Table 1 and 2). Of the 1,493 patients who presented with suspected acute appendicitis, 51%, 95% CI [48.5,53.5] were admitted for observation or for surgery. Of these, 62%, 95% CI [58.5,65.5] went to the OR for an appendectomy and 91%, 95% CI [88.4,93.6] were shown to have had pathology-proven appendicitis (Figure 2). None of the 739 children sent home returned to this hospital within the subsequent two weeks and diagnosed with acute appendicitis and were therefore presumed to not have appendicitis. Of the 430 with pathology-proven appendicitis, 23%, 95% CI [19.0,94.9] had a CT while 57%, 95% CI [52.3,61.7] had an abdominal US.

**Figure 3:**
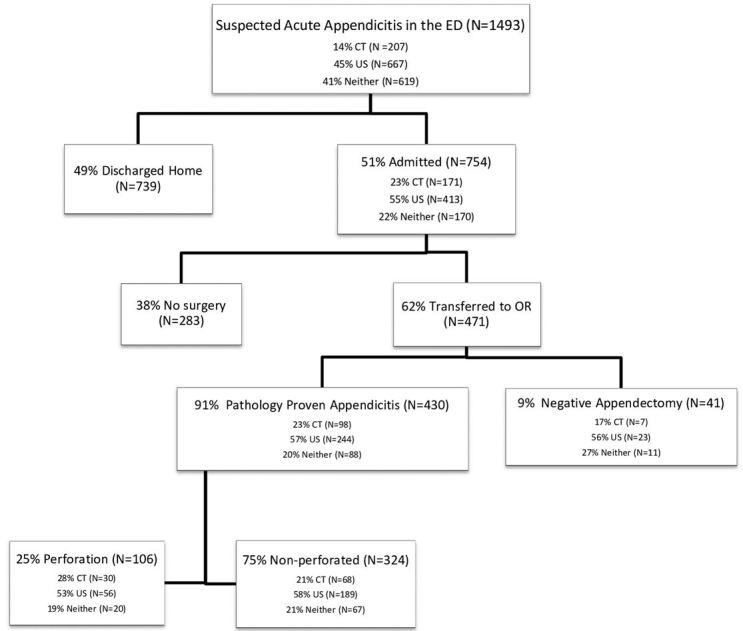
Flow diagram of initial ED presentation to discharge diagnosis and disposition from hospital. *ED,* emergency department; *CT,* computed tomography; *US,* ultrasound; *OR,* operating room

**Figure 4:**
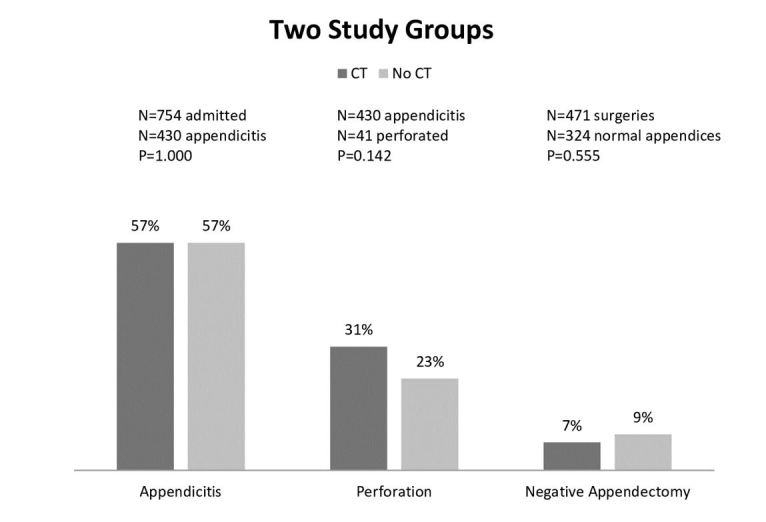
This figure shows the rate comparison of the two study groups and the three outcomes: pathology proven appendicitis, rupture, and negative appendectomy. *CT,* computed tomography

The frequency of pathology-proven appendicitis was similar for children who had a CT (57 %, 95% CI [49.6,64.4]), compared to those without a CT (57%, 95% CI [52.9,61.0]) (p=1.00) (Figure 3). The frequency of pathology-proven appendicitis was similar for children who received a CT (57%, 95% CI [52.3,61.7]), or an abdominal US (59%, 95% CI [55.0,60.1]), or those who received neither (53%, 95% CI [49.2,56.8]) (p=0.39). Of the 107 patients found to have a perforated appendix, 28%, 95% CI [19.5,36.5] of them had undergone pre-operative CT. The rate of perforation was similar for children who had a pre-operative CT (18%, 95% CI [12.2,13.8]) compared to those who did not (13%, 95% CI [10.2,15.8]) (p=0.15). No significant difference emerged when the three study groups were compared for rate of perforation; CT (31%, 95% CI [26.6,35.4]), US (20%, 95% CI [16.2,23.8]) and neither (29%, 95% CI [24.7,33.2]) (p=0.07) (Figure 4). 9%, 95% CI [6.4,11.6–2.58] of patients were determined to have a negative appendectomy and 17%, 95% CI [5.5,28.5] had a CT scan. The rate of negative appendectomy was not significantly different for children who had a pre-operative CT (7%, 95% CI [2.1,11.9]) versus those who did not (9%, 95% CI [6.0,11.9]) (p=0.56). The proportion of children who went to the OR and had a negative appendectomy was similar for those with CT (7%, 95% CI [2.1,11.9]), those with US (8%, 95% CI [4.7,11.3]) and those with neither (12%, 95% CI [5.9,18.1]) (p=0.44).

Of the 754 patients who were admitted to inpatient units, 283 did not undergo surgery; only six of those patients had abdominal CTs interpreted as positive for appendicitis by a radiologist at a community hospital that were later determined to be negative when read by a pediatric radiologist. Eleven of the patients admitted returned to the ED within seven days of their initial ED admission. Of these 11, four had abdominal CT interpretations that were negative for appendicitis and were admitted for inpatient observation. These patients had a discharge diagnosis of “RLQ Abdominal Pain” or “Abdominal Pain Site NOS,” as indicated by the ICD-9 code. The remaining seven patients who returned did not have abdominal CTs during their initial ED visit; two were discharged home from the ED with a diagnosis of acute gastroenteritis; two were discharged home and one was admitted for observation with a diagnosis of abdominal pain; one was discharged home with a diagnosis of ovarian cyst; and one had a discharge diagnosis of mesenteric adenitis. Table 3 compares false positive rates (FPR) and false negative rates (FNR) for US and CT.

Community hospitals performed the majority (61%) of abdominal CTs whereas the most (89%) abdominal USs were done at a children’s hospital.

## DISCUSSION

The objective of this study was to determine if diagnostic imaging was associated with clinical outcomes (e.g., pathology-proven appendicitis, rate of perforation or negative appendectomy) for children who present to a pediatric ED with suspected acute appendicitis. The results of this study demonstrated that patients who underwent a pre-operative abdominal CT were equally as likely to have pathology-proven appendicitis, perforated appendices, or negative appendectomies when compared to patients who did not undergo CT pre-operatively. Additionally, negative appendectomy and perforation rates were similar for children who received either a diagnostic CT or US and children without any diagnostic imaging. Riesenman and colleagues reported no difference in perforation rates for children with and without diagnostic CT studies but an increased length of stay for children who had a CT.^22^ Other authors have reported similar findings that despite an increased use of diagnostic imaging, there have not been any significant decreases in rates of negative appendectomy and perforation.^17^–^19^, ^23^

Because the use of CTs has been increasing at an approximate rate of 10% each year,^11^,^24^ more attention has been given to the risks involved with radiation exposure from CT.^10^–^14^,^25^ The amount of ionizing radiation from one CT study is approximately 100 times that of a plain radiograph, which may increase the potential to induce malignant cell divisions.^25^ Staged diagnostic protocols and scoring algorithms have been explored by several investigators who report that implementation of the protocol or scoring system not only reduced radiation exposure but also was found to have a high specificity and sensitivity when used to diagnose appendicitis.^5^,^6^,^26^ Moreover, Kim et al. studied the effects of lowering radiation doses involved with CTs used to diagnose appendicitis and found that negative appendectomy rates were similar in patient groups who received the low dose or standard dose CTs. Sensitivity and specificity in diagnosing appendicitis and perforated appendicitis were not found to be significantly different in Kim’s study.^27^

In this study, children who had pre-operative CTs were more inclined to have a perforated appendix (31%) at the time of surgery, although this was not statistically significant. The increase in perforation rate in children who had a CT may be attributable to a delay in seeking care, or a delay in surgery or transfer as a result of having a CT.^9^ Other potential factors that may have influenced the clinician to order a CT include concerning symptoms for perforated appendix, longer latency time to presenting to the ED, and/or equivocal US studies.^8^, ^28^

This study found that the majority (61%) of patients had abdominal CTs at community hospitals, whereas only 11% of patients had abdominal USs at a community hospital. Recent publications have reported positive correlations between the number of diagnostic CTs performed and community hospitals.^28^, ^29^ In this study, six patients had a positive CT interpreted at a community hospital that were later determined to be negative for appendicitis when interpreted by a children’s hospital radiologist. A recent study by Saito et al. reported that children evaluated for appendicitis at community hospitals were less likely to have an abdominal US and diagnostic accuracy of both CT and abdominal USs was reduced if the imaging was performed and interpreted at a community hospital.^29^ A potential explanation for these observations might be due to a lack of US availability and adequately trained US technicians at community hospitals, especially at night.^30^

Using US as the primary imaging modality in children with suspected acute appendicitis has been shown to be cost effective and reduces the number of CTs ordered.^5^,^6^,^26^,^31^ With the cost of an abdominal CT estimated to be triple the cost of an abdominal US^32^ it may be appropriate to employ US initially. This approach will not only keep costs down but will prevent some children from being exposed to harmful radiation. Pediatric surgeons at our institution are willing to operate on children with suspected appendicitis who have classic history, exam and laboratory findings, but when diagnostic uncertainty exists US is used.

A prospective, multi-center study to better identify how and when to use CTs in children may decrease radiation exposure while ensuring good clinical outcomes.

Because the rate of complications is similar and CT carries the added risk of radiation, we believe that the use of CT should be reserved for children who pose diagnostic challenges or risks of other pathologies.

## LIMITATIONS

This is a retrospective, single-center non-randomized study, where imaging decisions were left to the treating physician as described in the methods section. The initial definition used to identify children who presented to the ED with suspected acute appendicitis may have limited our study population. It is possible that some children with appendicitis were missed. Additionally, follow-up contact on the 739 patients discharged from the ED was not possible, and patients who were discharged with a diagnosis other than appendicitis may have presented to another institution and ultimately been diagnosed with appendicitis. However, in our region it is rare for a community hospital to perform an appendectomy on a child. Only 11 children returned to our ED within seven days of original presentation, and none had appendicitis. Also, no children who were originally sent home returned to this study hospital within two weeks of the original visit and diagnosed with acute appendicitis. The availability of US in community hospitals may limit its use and decrease the generalizability of our results. US was available in the study hospital weekdays from 7:30am until 11:00p, and Saturday mornings. US was not available overnight or on Sundays. Propensity score analysis was not done because details regarding specific surgeon and illness severity on presentation were not available.

## CONCLUSION

The rate of complications did not vary significantly for children with suspected acute appendicitis who had CT versus US, in conjunction with surgical consult. The proportion of children with pathology-proven appendicitis, ruptured appendices, and negative appendectomy was similar for children regardless of type of imaging used.

## Figures and Tables

**Figure 5:**
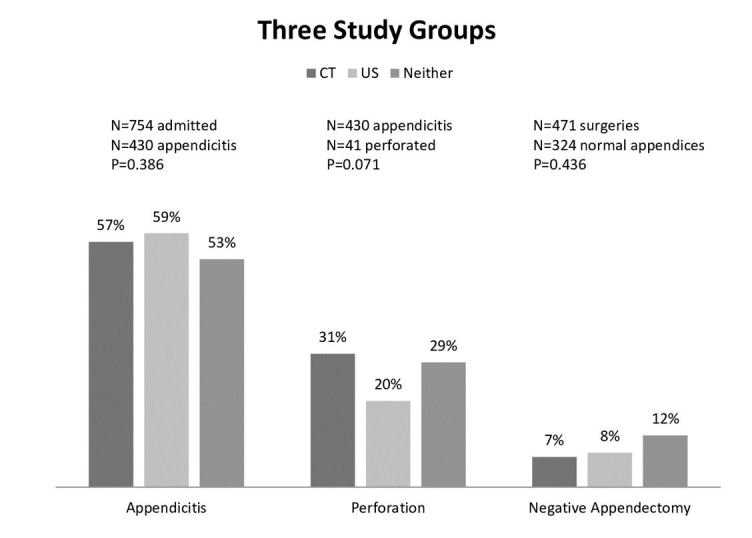
This figure shows the rate comparison of the three study groups and the three outcomes: pathology proven appendicitis, rupture, and negative appendectomy. *CT,* computed tomography; *US,* ultrasound

**Table 1:** Demographic table comparing the two study groups: children who had a diagnostic computed tomography (CT), and those who did not have a CT.

	CT 16% (N=233)	No CT 84% (N=1260)
Age (years)	11.9 (SD 3.6)	10.8 (SD 4.0)
Gender (% female)	50%	50%
Race		
White	57.1%	52.9%
Hispanic	22.3%	25.3%
Black	7.7%	9.9%
Other	12.4%	11.8%
Insurance		
Private	58.7%	56.7%
Public	39.1%	37.4%
Self-pay	2.2%	3.7%

**Table 2:** Demographic table comparing the three study groups: children who had a diagnostic computed tomography (CT), children who had a diagnostic ultrasound (US), and those who had neither.

	CT 16% (N=233)	US 51% (N=766)	Neither 33% (N=494)
Age (years)	11.9 (SD 3.63)	11.0 (SD 4.05)	10.5 (SD 4.00)
Gender (% female)	50%	54%	44%
Race			
White	57.5%	57.3%	46.0%
Hispanic	22.3%	23.4%	28.1%
Black	7.7%	7.8%	13.2%
Other	12.4%	11.3%	12.6%
Insurance			
Private	58.7%	59.5%	55.5%
Public	39.1%	37.1%	40.1%
Self-pay	2.0%	3.5%	4.3%

**Table 3:** Comparison of false positive rate (FPR) and false negative rate (FNR) for computed tomography (CT) and ultrasound (US) for the 754 patients who were admitted.

	CT 20% (N=150)	US 53% (N=397)
FPR	12%	16%
FNR	16%	23%
